# Anti-Cancerous Potential of Polysaccharide Fractions Extracted from Peony Seed Dreg on Various Human Cancer Cell Lines Via Cell Cycle Arrest and Apoptosis

**DOI:** 10.3389/fphar.2017.00102

**Published:** 2017-03-03

**Authors:** Fang Zhang, Jun-Jun Shi, Kiran Thakur, Fei Hu, Jian-Guo Zhang, Zhao-Jun Wei

**Affiliations:** School of Food Science and Engineering, Hefei University of TechnologyHefei, China

**Keywords:** polysaccharides, peony seed dreg, anti-cancerous, cell cycle, apoptosis, molecular mechanisms

## Abstract

In this study, four homo/heterogenous polysaccharides (HBSS, CHSS, DASS, and CASS) extracted from peony seed dreg with respective molecular weights of 3467, 4677, 229, and 56 kDa were evaluated for anti-cancerous attributes in prostate cancer cells (Pc-3), colon cancer cells (HCT-116), human breast cancer cells (MCF-7), cervical cancer (Hela cells) and human embryonic kidney 293 (HEK 293) cells as control. Among them, CASS and DASS extracted by alkali, consisted of 34.43% *Gal*, 26.39% *Ara*, 21.80% *Glc* and 35.77% *Ara*, 19.35% *Gal*, 17.77% *Man*, respectively. CASS fraction had the most significant inhibitory effects on all the cell lines used whereas HBSS had least effect. The CASS shown remarkable inhibition and cytotoxic effects in Hela cells followed by other cell lines as compared to 5-fluorouracil (5-FU). CASS arrested cell cycle in G0/G1 phase except MCF-7 cells and increased apoptotic cells percentage varied in different treated cells. CASS down regulated the expression of Cyclin A/B1/D1/E1, CDK-1/2/4/6 and p15/16/21/27 excluding p53. The notable change in expression of proteins (Cytochrome C, Bax, Bcl-2, p-Caspase-3, -8, -9, and PARP) was observed followed by Apaf-1 and Survivin. These findings indicated that CASS has an anti-cancerous potential in the treatment of human cancers which make it a potent candidate in functional foods.

## Introduction

In the current decade, cancer has still remained the top listed death-causing disease despite of developments in tools of diagnosis, treatment and prevention and it is the most common-death causing disease (Razali et al., 2016). Cancer occurs when the cells proliferate without any control. The low efficacy of currently available drugs to cure cancer demands the identification of natural compounds for cancer prevention (Ale et al., 2011). Therefore, search for effective anti-cancerous drugs with low toxicity is necessary for its treatment. Most of the anti-cancerous drugs currently used in chemotherapy are cytotoxic to normal cells (Fan et al., 2012). The emergence of new anti-cancerous agents with fewer side effects has become an essential goal in cancer chemotherapy. Natural phytochemicals in medicinal herbs are one the most attractive strategies in cancer chemotherapy (Miao et al., 2010).

There are recent reports for anti-cancerous activities of various polysaccharides form different sources (Meng et al., 2016). Recently, there is a shift from microbial polysaccharides to plant polysaccharides because latter are mainly non-toxic and may not cause side effects (Schepetkin and Quinn, 2006). Plant polysaccharides from different sources have long been studied and widely used for a variety of purposes including food, animal feed, medicine, and papermaking (Liu et al., 2015). These natural polysaccharides have drawn much attention in recent years due to array of biological activities that include antioxidant, immuno-modulatory, anti-tumor, gastrointestinal protection, anti-diabetic, and hepatoprotective effects (Li N. et al., 2012; Cho et al., 2015; Wang et al., 2015; Shi et al., 2016a,b).

Peony, a national plant with multifaceted properties and diverse uses, is widely cultivated in the other parts of the world including Japan, Korea, New Zealand, Europe, and North America (Zhou et al., 2014). The four members of peony seed dreg polysaccharides displayed various different in thermal, emulsifying and anti-oxidant properties, which are important for peony seed dreg in potential industrial applications of functional foods (Shi et al., 2016b).

The recent report has focused on signaling molecules to demonstrate the efficacy of polysaccharides in the induction of cytotoxicity to cancer cells (Alwarsamy et al., 2016). Generally, polysaccharide work via two mechanisms against tumor cells: direct action (inhibition of tumor cell growth and apoptosis induction) and indirect action (immunostimulation). Besides the indirect action, several polysaccharides have shown direct effects on cancer cells. Many in-vitro and in-vivo studies suggested that inhibition of tumor cell proliferation and/or induce their death by apoptosis after treatment with polysaccharides (Ooi and Liu, 2000; Bao et al., 2001; Smith et al., 2002; Tao et al., 2006; Zhang et al., 2007; Fan et al., 2014). Moreover, anticancer properties of biologically active compounds isolated from mushrooms are mostly attributed to polysaccharides through various studies (Wasser and Weis, 1999; Wasser, 2002). Although a few polysaccharides with direct cytotoxic effects not through the immune system mediation have been reported (Matsuda et al., 2003; Leon et al., 2004) but their action mechanism is still not clear and needs further research.

The important process of the programmed cell death is cell cycle arrest and apoptosis, which is mostly regulated by cell growth signaling molecules. There are various studies which reported the role of polysaccharides in cell cycle induction, inhibition of cell proliferation, induction of apoptosis and targeted the regulatory pathways and mechanisms of cell cycle genes and their protein products by using different cancer cell lines (Chu et al., 2016; Guo et al., 2016; Liu G. et al., 2016; Sun et al., 2016; Yang et al., 2016; Zhang et al., 2016). Although the anti-cancerous activity of various plant polysaccharides on different types of cancer cells have been revealed, however, the principal active ingredients in these extracts and the exact underlying mechanisms still remain largely unknown so far. A recent review has reported the anti-cancerous mechanism of *Gynostemma pentaphyllum* (Thunb.) Makino *(Jiaogulan)* GPM polysaccharides through cell cycle arrest (modulation of several cell cycle regulatory proteins (CDK-2, CDK-4, and CDK-6) (Chen et al., 2009; Cheng et al., 2011), induction of apoptosis (regulation of anti-apoptotic proteins Bcl-2, Bcl-xl and pro-apoptotic proteins Bax, Bad, and Bak (Wang et al., 2002; Hsu et al., 2011; Lin et al., 2011; Lu et al., 2012), glycolysis inhibition and immunomodulation.

Though, data on the effects of peony seed polysaccharides on cancer cells are still not present. To the best of our knowledge, our study is the first ever report for anti-cancerous attributes of extracted polysaccharides from peony seeds dreg. The previously extracted four polysaccharides (HBSS, CHSS, DASS, and CASS) (Shi et al., 2016a) in our laboratory were subjected for molecular weight determination, their chemical composition analysis and further examined for their effects on inhibition of cell proliferation, cell cycle arrest, induction of apoptosis. Additionally, their role in regulatory pathways and mechanisms of cell cycle genes expression and their protein products by using different cancer cell lines such as prostate cancer cells (Pc-3), colon cancer cells (HCT-116), human breast cancer cells (MCF-7), cervical cancer (Hela cells) and human embryonic kidney 293 (HEK 293) cells (control) were evaluated in the current study.

## Materials and Methods

### Materials

The obtained polysaccharide fractions (HBSS, CHSS, DASS and CASS) (Supplementary Figure S1) from our previous study (Shi et al., 2016a) were analyzed for further experiments. Standard monosaccharides (D-glucose (*Glc*), D-mannose (*Man*), D-xylose (*Xyl*), L-galactose (*Gal*), L-rhamnose (*Rha*), and L-arabinose (*Ara*)) were purchased from Sigma–Aldrich Co., Ltd. (St. Louis, MO, USA). McCoy’s 5A Modified Medium (McCoy’s 5A), Roswell Park Memorial Institute Medium (RPMI 1640), Dulbecco’s Modified Eagle’s Medium (DMEM), FBS, L-glutamine, penicillin-streptomycin and 0.25% trypsin solution without EDTA were procured from Invitrogen (Carlsbad, USA). The cell lines (Hela, Pc-3, MCF-7, HCT-116, and HEK293) were purchased from Shanghai wei atlas biological technology co., LTD.

### Chemical Composition Analysis

#### Sugar Composition

The monosaccharide composition of polysaccharide fractions was analyzed by Gas Chromatography (GC; model-8700, Perkin-Elme). Samples (5 mg) were hydrolyzed by 4 mL 2 M trifluoroacetic acid (TFA) at 120°C for 4 h in a sealed glass tube. The residual TFA was removed using a rotary vacuum evaporator and the monosaccharides were reduced with 30 mg NaBH_4_ for 3 h at room temperature after dissolved in distilled water. The reaction solution was neutralized with 25% CH_3_COOH until there were no air bubbles. After removing the water phase, the residue was allowed to react with 3 mL of acetic anhydride and 3 mL of pyridine for 1 h. The reaction products were alditol acetate derivatives and analyzed by GC. The operation method used was reported in the previous study (Wang J.-H. et al., 2010).

#### Uronic Acid Content

The content of UA was estimated using galacturonic acid as the standard. Polysaccharide solution (0.25 mL, 2 mg/mL) was mixed with 1.5 mL of disodium tetraborate sulfuric acid solution (12.5 mM) and the mixture was incubated in boiling water-bath for 5 min. After cooling in ice bath, 25 μL of mhydroxydiphenyl NaOH solution (0.15%) was added into the reaction mixture, and the absorbance at 520 nm was determined with a spectrophotometer. The UA content was calculated by substituting the absorbance into standard curve.

#### Protein Content

The content of protein was determined by the method of Bradford (Bradford, 1976) using bovine serum albumin as standard. Polysaccharide solution (0.5 mL, 2 mg/mL) was mixed with 2.5 mL protein reagent (50 mL of 2.0 mg/mL Coomassie Brilliant Blue G-250 95% ethanol solution with 100 mL 85% phosphoric acid). After agitating, the Abs at 595 nm was determined with a spectrophotometer. The protein content was calculated by substituting the Abs into standard curve.

#### Carbohydrate Content

The content of total carbohydrate was measured by the phenol–sulphuric acid method using glucose as standard (Dubois et al., 1956). Polysaccharide solution (0.5 mL, 100 μg/mL) was mixed with 0.5 mL phenol solution (6%) and 2.5 mL sulfuric acid. After 20 min, the Abs at 490 nm was determined with a spectrophotometer. The carbohydrate content was calculated by substituting Abs into standard curve.

### Determination of Molecular Weight

The molecular weight of polysaccharide fractions was determined by high-performance gel permeation chromatography (HP-GPC) using a High-Performance Liquid Chromatography (HPLC) system (Model LC 1100, Agilent Co., USA) equipped with a TSK-gel column (Model G4000 PWXL, Tosoh Co., Japan) and a differential refractive index detector. Twenty microliter of sample solution (5.0 mg/mL) was injected in each run with ultrapure water containing 0.1% (w/w) NaN_3_ as the mobile phase, while the flow rate was 0.5 mL/min. Standard dextran with molecular weights of 10.0, 20.0, 40.0, 110.0, 500.0, and 2000.0 kDa were passed through the column under the same experimental condition. Then a calibration curve (log molecular weight-retention time) of molecular weight of standard was obtained.

### Cell Culture

The prostate cancer cells (Pc-3), colon cancer cells (HCT-116), human breast cancer cells (MCF-7), cervical cancer cells (Hela) and human embryonic kidney 293 cells (HEK293) were grown in monolayer with McCoy’s 5A, RPMI-1640 or DMEM media supplemented with 10% (v/v) heat-inactivated FBS, 2 mM L-glutamine, and 1% penicillin-streptomycin in a humidified atmosphere containing 5% CO_2_ (Wang et al., 2012).

### The Inhibitory Effect of Polysaccharide Fractions on Cells

The four types of polysaccharides were investigated for their effects on cancer cell proliferation by CCK-8 (DOJINDO Corp., Japan) colorimetric method. Briefly, 2 × 10^5^ cells were seeded in each well of 96-well plates with 100 μL growth medium and after overnight incubation, four types of polysaccharides by serial concentrations (50, 100, 200, 300, 400, and 500 μg/mL) were further added for 48 h followed by replacing old medium with 100 μL fresh medium containing 10 μL CCK-8 solution. Further, after incubation at 37°C for 2.5 h, the plate was read at 450 nm using a spectrophotometric plate reader (BioTek Instruments, Inc., Winooski, VT, USA).

Inhibition ability was expressed as the percentage of absorbance in the treated cells compared to negative control:

Cell inhibition ability (%) = (OD negative control – OD treatment)/(OD standard – OD blank) × 100%.

The inhibition rate of polysaccharide fractions on the cell lines can be evaluated as follow:

(1) $$\text{y=}\frac{{\text{A}}_{\text{1}}{\text{-A}}_{\text{2}}}{{\text{1+e}}^{({\text{x-x}}_{\text{0}})\text{/dx}}}{\text{+A}}_{\text{2}}$$

### The Cytotoxic Effect of Polysaccharide Fractions

Cells were cultured at the density of 2 × 10^5^ cells per well in 96-well plates for 10 h, and were transferred into the medium containing polysaccharide fractions at different concentrations (50, 100, 200, 300, 400, and 500 μg/mL). After 48 h, the culture media were collected and LDH cytotoxicity was determined by a microplate reader at 490 nm using the cytotoxicity LDH assay kit (Dojindo, Japan).

### Effect of Polysaccharide Fractions on Cell Cycle

Cells of log phase were treated with 200 μg/mL of CASS fraction. The treated cells were digested and washed with PBS, then immobilized with chilled 70% ethyl alcohol and re-washed with PBS, and then, finally re-suspended with PI. Cells were incubated at 4°C in darkness for 30 min. The distribution of cells in various cell cycle phases was detected on flow cytometer (Becton Dickinson, San Diego, CA, USA) as reported by previous report (Li et al., 2016). Proportion of the cells in G0/G1, S, and G2/M phases were analyzed by the Flowjo software.

### Effect of Polysaccharide Fractions on Cell Apoptosis

The flow cytometry was used as previously described by Wen et al. (2015) with little modifications (Wen et al., 2015). As described earlier, four types of cell lines were seeded in six-well microplates, after incubation for 10 h, the cells were treated with CASS fraction (200 μg/mL) for another 24 h, and then, the cells were harvested by trypsinization. After centrifugation at 1000 rpm for 5 min, the cells were washed with chilled PBS, and re-suspended in 400 L of binding buffer containing 5 μL of annexin V-FITC and 10 μL of PI, and then analyzed by a BD FACS Verse flow cytometer FACS calibur Flow Cytometer (Becton Dickinson).

### Quantification of mRNA Expression of Cell Cycle Genes by qRT-PCR in Hela Cells

In our study, to evaluate the mRNA level of cell cycle genes, total RNA was extracted using Trizol Reagent (Invitrogen, Life Technologies, USA) followed by first-strand cDNA synthesis using the Prime Script 1st Strand cDNA Synthesis kit and the Oligo dT-adaptor primer in a series of standard 10 μL reverse transcription reactions. Changes in the expression levels of mRNA in Cyclin A/B1/D1/E1, CDK-1/2/4/6, p15/16/21/27, and p53 were evaluated by reverse-transcription PCR (RT-qPCR), which was carried out using EvaGreen Master Mix (Biotium, Hayward, CA, USA). The primers used in our study are presented in **Table 1**. RT-qPCR was performed using the ABI Step One Plus system (Applied Biosystems) followed by melting curve analysis with the following cycling program: initial activation at 95°C for 3 min, followed by 40 cycles of denaturation at 95°C for 10 s, annealing at 60°C for 20 s. GAPDH served as a control for sample loading and integrity.

**Table 1:** Yield, composition of polysaccharides from peony seed dreg.

Fraction	Yield (g/100 g AIS)	Rha	Ara	Xyl	Man	Glc	Gal	UA	Carbohydrate content	Protein content
HBSS	10.265	5.07	3.60	1.07	55.74	33.93	0.59	2.20	88.90	8.445
CHSS	4.247	1.05	11.27	0.47	2.18	2.42	82.61	0.14	84.67	6.305
DASS	8.574	13.33	35.77	1.03	17.77	12.75	19.35	1.44	80.38	9.685
CASS	4.693	3.39	26.39	11.80	2.19	21.80	34.43	0.40	85.45	4.985

*Rha, rhamnose; Ara, arabinose; Xyl, xylose; Man, mannose; Glc, glucose; Gal, galactose; UA, uronic acid.*

### Western Blot Analysis

Weston blot was carried out according to Czemplik et al. (2016) with some modifications. For analysis of cell cycle related proteins, following the treatment with CASS (100, 300, and 500 μg/mL) for 48 h, Hela cells were washed 3 times in chilled PBS and lysed with an ice-cold radioimmunoprecipitation (RIPA) buffer containing a protein phosphatase inhibitor and a complete protease inhibitor mixture for 30 min over ice. To obtain the cytosol fraction, the cell lysates were centrifuged at 15,000 r/min for 15 min at 4°C. In brief, the cytosolic proteins were boiled in a loading buffer, followed by denaturation of proteins which were separated by sodium dodecyl sulfate-polyacrylamide (SDSP) gel electrophoresis and transferred to a 0.45 μm polyvinylidene difluoride (PVDF) membrane. After 2 h at room temperature of incubation in a blocking buffer (150 mM NaCl, 20 mM Tris-HCl, 0.1% Tween 20, and 5% defat milk), the membranes were incubated with the specific primary antibodies overnight at 4°C. Subsequently, the blot was washed 3 times with Tris Buffered Saline Tween (TBST) (150 mM NaCl, 20 mM Tris-HCl, and 0.1% Tween 20), followed by incubated with the appropriate secondary antibody for 3 h (Yan et al., 2017).

### Statistical Analysis

Statistical analysis was carried out by using SPSS 18.0 software. All the data were expressed as mean ± SD (*n* ≥ 3).

## Results

### Chemical Properties of Polysaccharide Fractions

**FIGURE 1:**
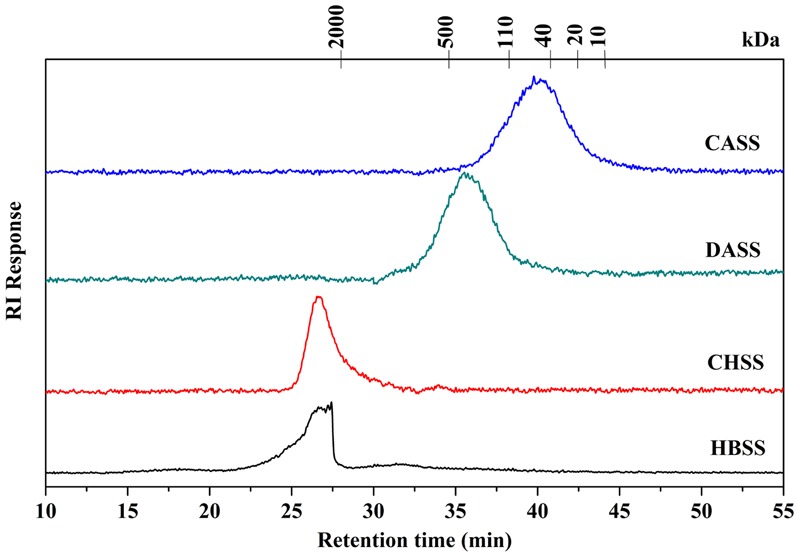
**Determination of molecular weight of different fractions of polysaccharides (HBSS, CHSS, DASS, and CASS)**.

The chemical composition of polysaccharide fractions, including total carbohydrate, protein, UA content and monosaccharide composition were summarized in **Table 2**. The total carbohydrate content of the four fractions (HBSS, CHSS, DASS, and CASS) were determined to be 88.90, 84.67, 80.38, and 85.45%, respectively. In addition, small amounts of protein found in HBSS, CHSS, DASS, and CASS were 8.44%, 6.30%, 9.69% and 4.99%, respectively. Their uronic acid content were 2.20, 0.14, 1.44, and 0.40%, respectively. The respective values obtained from GC analysis corresponded to rhamnose (*Rha*), arabinose (*Ara*), xylose (*Xyl*), mannose (*Man*), glucose (*Glc*), and galactose (*Gal*), respectively, by matching their retention time with those of monosaccharide standards under the same analytical conditions (Supplementary Figure S2). Our results indicated that the fractions (CASS and DASS) obtained by alkali methods were of heterogenous nature where CASS fraction composed 34.43% *Gal*, 26.39% *Ara* and 21.80% *Glc* and DASS consisted of 35.77% *Ara*, 19.35% *Gal* and 17.77% *Man* as compared to other two fractions. From the HPLC results (**Figure 1**), the single peaks for polysaccharide fractions were obtained, confirming the four fractions were homogeneous polysaccharides. Based on the calibration curve, Log Mw = –0.14531 T + 7.52584 (*R*^2^ = 0.9684) (where Mw was the molecular weight and the *T* presented the retention time), the average molecular weights of these fractions were calculated as 3467.37, 4677.35, 229.09, and 56.23 kDa, respectively.

**Table 2:** Primers for real-time PCR.

Gene	Primer	Sequence (5′–3′)
Cyclin A	Forward	AGACTGAGTGGTTGGATGGCA
	Reverse	TGTCCACAGTCAGCAATGGTG
Cyclin B1	Forward	AAAGGCGTAACTCGAATGGA
	Reverse	CCGACCTTTTATTGAAGAGCA
Cyclin D1	Forward	ATGGAACACCAGCTCCTGTGCTGC
	Reverse	TCAGATGTCCACGTCCCGCACGT
Cyclin E1	Forward	GGATTATTGCACCATCCAGAGGCT
	Reverse	CTTGTGTCGCCATATACCGGTCAA
CDK-1	Forward	TCCGCAACAGGGAAGAAC
	Reverse	GAGCCTTTTTAGATGGCTGCT
CDK-2	Forward	CTTTGGAGTCCCTGTCCGTA
	Reverse	CGAAAGATCCGGAAGAGTTG
CDK-4	Forward	TGCACAGTGTCACGAACAGA
	Reverse	ACCTCGGAGAAGCTGAAACA
CDK-6	Forward	CATCGTTCACCGAGATCTGA
	Reverse	CCAACACTCCACATGTCCAC
P15	Forward	GCGGCAGCTCCTGGAAG
	Reverse	GGGTCGGCACAGTTGG
P16	Forward	CTTCCTGGACACGCTGGT
	Reverse	ATCTATGCGGGCATGGTTACT
p21	Forward	CCACAGCGATATCCAGACATTC
	Reverse	GAAGTCAAAGTTCCACCGTTCTC
p27	Forward	AGCGACCTGCTGCAGAAGAT
	Reverse	TTACGTCTGGCGTCGAAGGC
P53	Forward	GTCAGAAGCACCCAGGACTT
	Reverse	CTCCCAAACATCCCTCACAG
GAPDH	Forward	TGCCCTCAACGACCACTTTG
	Reverse	TACTCCTTGGAGGCCATGTG

### The Inhibitory Effect of Polysaccharides Fractions on Cell Proliferation

**FIGURE 2:**
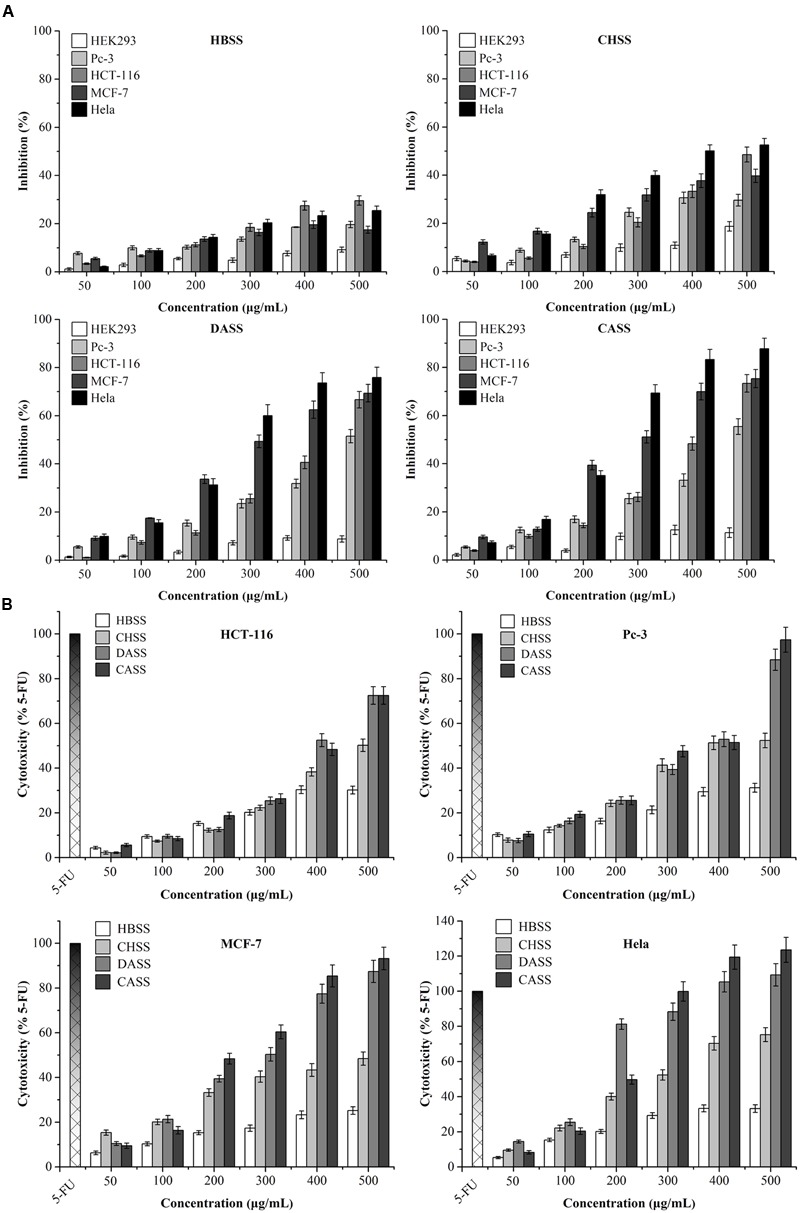
**The inhibition and cytotoxicity assessment of polysaccharides on different cancer cell lines. (A)** Inhibitory effects of polysaccharides on the growth of treated cells; **(B)** the cytotoxicity of polysaccharides and 5-FU on treated cells. All the data were collected after treatment for 48 h, and expressed as mean ± SD of three replicates.

To assess the anti-cancerous potential of different fractions for cancer cells, the results showed that CASS and DASS had significantly inhibited the cell proliferation followed by CHSS and HBSS fractions (**Figure 2A**). In particular, CASS fraction had the most significant inhibitory effects on all the cell lines used whereas HBSS had least effect. All the fractions showed the inhibition in a concentration dependent manner with a varied effect on each cell lines. When cells were treated with doses higher than 300 μg/mL of CASS for 48 h, 69% of Hela cells were inhibited compared to control (**Figure 2A**). These results suggested different extraction methods might have significant effect on biological activities of polysaccharide fractions as well as the inhibition trait varied with cell line strains and caused high cytotoxicity at high doses.

### Cytotoxic Effects of Polysaccharide Fractions on Cancer Cells

The release of LDH is an index to assess the integrity of cell membranes and also to evaluate the efficacy of cytotoxicity on cancer cells by plant derived extractions. As shown in **Figure 2B**, after exposure to different polysaccharide fractions for 48 h, the LDH vitalities were quantitatively assessed for the cytotoxicity on treated cells and compared with 5-FU under different concentrations (25 Mm for HCT-116 and MCF-7 cells and 50 μM for Pc-3 and Hela cells). For LDH activity, CASS has shown the most significant effects on different cancer cell lines as compared to remaining three fractions in a concentration dependent manner. Our results clearly displayed that CASS could be used as a potential anti-cancerous agent in medical research because it had shown remarkable cytotoxic effect at 300 μg/mL in Hela cells followed by other cell lines, such as Pc-3 and MCF-7 with increasing concentrations. Therefore, the potential of plant compounds for replacing chemotherapeutic drugs can be taken into consideration besides their safety and public health concerns in the future.

### Induction of Cell Cycle Arrest by Polysaccharide Fractions

**FIGURE 3:**
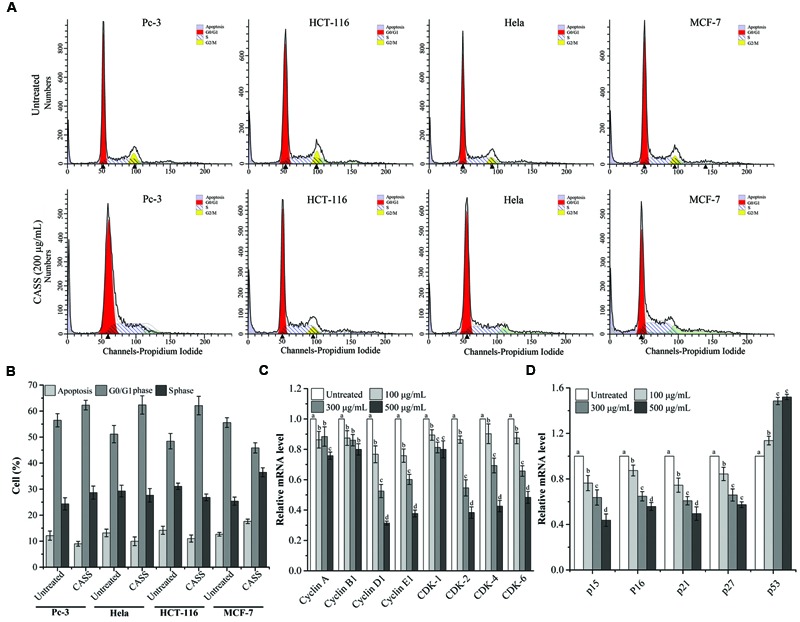
**(A)** CASS induces cell cycle arrest in treated cells. **(B)** The rate of cell cycle arrest in apoptosis and different phases. **(C,D)** The mRNA expression of Hela cell cycle related genes with increasing concentrations of CASS as compared to untreated cells. Relative gene-expression levels are expressed with GAPDH as an internal reference. Each value is presented as a mean ± standard deviation (*n* = 3). Values of a–d represent significantly different treatments within same gene, *P* < 0.05.

Flow cytometry results showed that CASS treatment for 24 h resulted in accumulation of all the cells in the G0/G1 phase except the MCF-7 cells which were arrested in S phase (**Figure 3A**). For HCT-116 cells, the G0/G1 phase reached upto 62.13% compared to the untreated cells (48.44%). On the other hand, the proportions of Pc-3 and Hela cells in G0/G1 phase evidently decreased from 56.48% and 51.16% to 62.30% and 62.37%, respectively. Whereas, the MCF-7 cells arrested in S phase with the numbers from 25.40 to 36.49% (**Figure 3B**).

### Induction of Apoptosis by Polysaccharide Fractions

**FIGURE 4:**
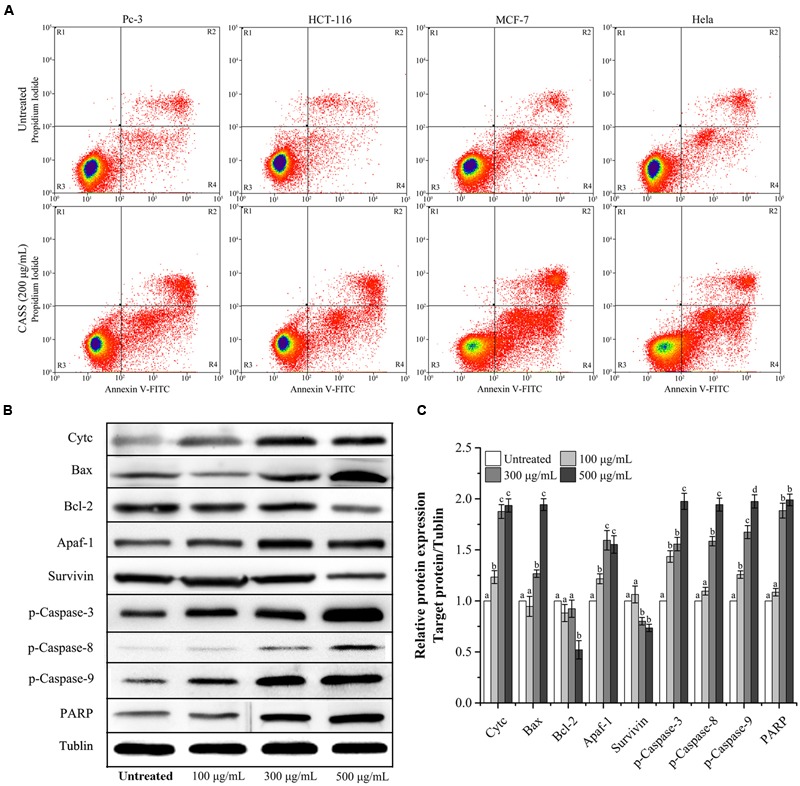
**(A)** The effects of CASS on treated cell apoptosis. **(B)** Effect of CASS on phosphorylation of apoptosis related proteins in treated Hela cells analyzed by western blot using tublin as an internal control. **(C)** The expression level of the targeted proteins with increasing concentrations of CASS as compared to untreated cells. Each value is presented as a mean ± standard deviation (*n* = 3). Values of a–d represent significantly different under different treatments within same gene, *P* < 0.05.

Considering that many cytotoxic agents can induce cell cycle arrest at different phases, then result in apoptosis (Gamet-Payrastre et al., 2000), we determined the occurrence of CASS induced apoptosis by PI and Annexin V-FITC staining methods. **Figure 4A** showed that the proportion of apoptotic cells significantly increased in cells treated with CASS (200 μg/mL for 48 h) upto 20.22% (Pc-3), 17.87% (HCT-116), 30.94% (Hela) and 38.73% (MCF-7), respectively.

### Anti-cancerous Mechanism of CASS on Hela Cells

According to our aforementioned data, CASS showed strong anti-cancerous effects, on the four kinds of cell lines used in our study in general and Hela cells in particular.

Further, Hela cells were given more preference to evaluate the mechanism responsible for cell cycle arrest and cell apoptosis by qRT-PCR and western blot. We evaluated the mRNA expression of molecular markers associated with G0/G1 and S phases (Cyclin A/B1/D1/E1, CDK-1/2/4/6, p15/16/21/27, and p53) and protein expression (Survivin, Cytochrome C, Bax, Bcl-2, Apaf-1, p-Caspase-3, -8, -9, and PARP). **Figures 3C,D** showed that expression of Cyclin D1/E1, CDK-2/4/6, p15/16/21/27 decreased with increasing concentrations of CASS followed by decrease in CDK-1, Cyclin A and B1. On the other hand, p53 expression effectively increased with increasing concentrations in treated cells. CASS also led to remarkable change in expression of proteins level (Cytochrome C, Bax, Bcl-2, p-Caspase-3, -8, -9, and PARP) compared with that in the control but did not affect significantly Apaf-1 and Survivin expression (**Figures 4B,C**).

## Discussion

The focus of recent research has been shifted to pharmacologically active natural polysaccharides because of their importance in a wide range of oncology studies as well as their non-toxic characteristics. Our polysaccharide fractions (HBSS, CHSS, DASS, and CASS) were subjected to series of experiments to evaluate their anti-cancerous effects. The molecular weight distribution pattern of CHSS showed only one fraction having a rather high molecular weight, while HBSS showed a broader molecular weight distribution representing populations with molecular weights lower than CHSS. Moreover, the molecular weight distribution pattern of the DASS is similar to that of CASS. The molecular weight of CASS fraction representing fractions with lowest molecular weight values than the other fractions. These trends observed in our study were in agreement with the previous reports for okra pods (Sengkhamparn et al., 2009) and blue berries (Hilz et al., 2005).

In general, high molecular weight contributes to anti-cancerous activity of plant polysaccharides, whereas reports also suggested that even low MW equally influence the anti-cancerous activities (Kashimoto et al., 2006). One study revealed that partially cellulose digested Aloe polysaccharide fractions within the size of 5–400 kDa had the most potent anti-cancerous activity, depicting the importance of size of polysaccharide for its biological activity (Im et al., 2005). It was reported that some low molecular weight polysaccharides, such as *schizophyllan*, present better antitumor activity against S-180 cells (Ooi and Liu, 2000). In addition, to trigger anti-cancerous events, the high percentage of proteins present could be of great importance. Because the larger polysaccharides possess more repeating units, thus increased variability infers higher molecular weight, and side chain proteins bind to receptors on the cell membrane surface which lead to strong cascade signals, therefore induce cell apoptosis (Gao et al., 1996). As based on seven antitumor polysaccharide-protein complexes of hetero-polysaccharides, with molecular weights ranging from 10 to 1000 kDa possessed remarkable anti-cancerous effects (Leung et al., 1997).

As known, anti-tumor activity of polysaccharides is partly dependent on its structure. Therefore, studies have proved that modified polysaccharides depicted altered structures and ultimately their anti-cancerous efficacy was observed to be enhanced (Wang J. et al., 2010; Li X.L. et al., 2012; Xu et al., 2016). Moreover, polysaccharide being as a macromolecular compound harbors complicated binding sites may lead to different biological activities of similar structured polysaccharides (Young and Jacobs, 1998; Zhang et al., 2000). In addition, various buffer treatments also resulted in changed anti-cancerous attributes of similar structured polysaccharides.

From our results, we can comprehend that; the alkali solution might alter the polysaccharide active sites and make them potent anti-cancerous agents. On the other hand, conventional extraction methods (water extraction or alcohol extraction) are mostly mild and help the obtained fractions to retain their molecular configuration and lead to weak anti-cancerous attributes. Moreover, the strong anti-cancerous activity of CASS could be due to its relatively small molecular weight, as confirmed with the previous reports (Im et al., 2005). Whereas, DASS might be because of their relatively high protein content, besides it can effectively bind with receptors on the cell membrane, and it also display an excellent solubility in water. The different extraction methods used in our previous study (Shi et al., 2016a) might have significant effect on biological activities of polysaccharide fractions as well as the inhibition trait varied with cell line strains and caused high cytotoxicity at high doses. Our results clearly indicated that CASS had shown remarkable cytotoxic effect at 300 μg/mL in Hela cells followed by other cell lines, such as Pc-3 and MCF-7 with increasing concentrations as compared to 5-FU. The Flow cytometry results showed that CASS treatment for 24 h resulted in accumulation of all the cells in the G0/G1 phase except the MCF-7 cells which was arrested in S phase. The proportion of apoptotic cells significantly increased in cells treated with CASS (200 μg/mL for 48 h) upto 20.22% (Pc-3), 17.87% (HCT-116), 30.94% (Hela), and 38.73% (MCF-7), respectively.

**FIGURE 5:**
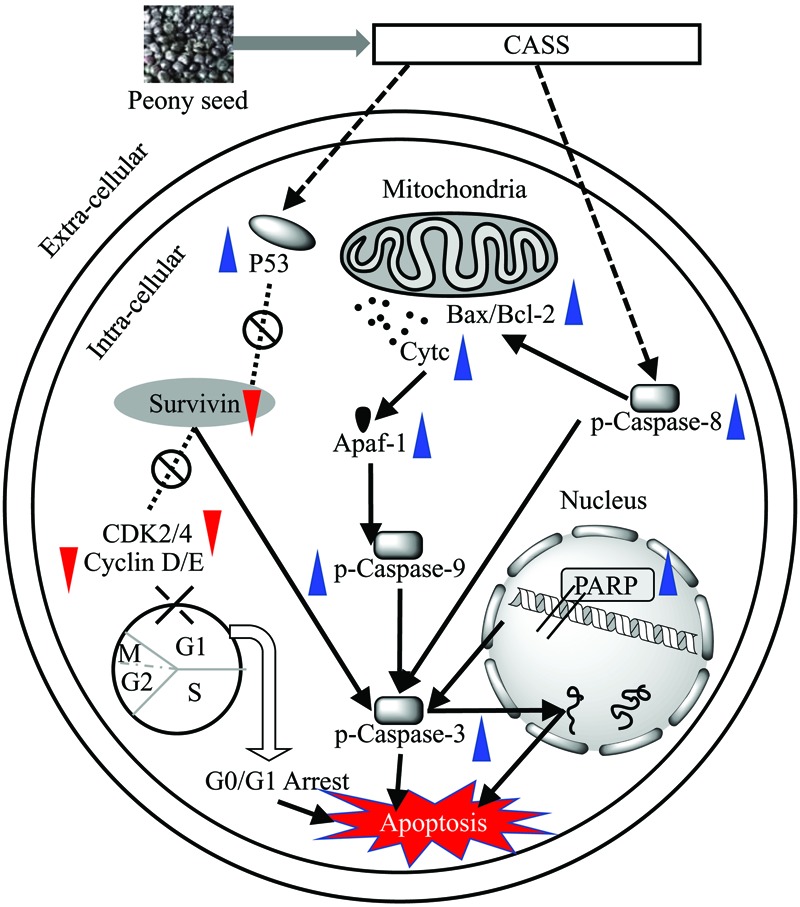
**Proposed mechanism for the effects of potent polysaccharide fraction (CASS) on cell cycle and apoptosis with a series of pathways involved in Hela cells**.

Besides, p53 a tumor suppressor gene involves in inhibition of all the proliferation via cell cycle arrest/cell apoptosis and responsible for more than half of human cancers (Levine, 1997; Willis and Chen, 2002). The mutations in p53 lead to uncontrolled cell proliferation and finally result in cancer. On the other hand, Cyclin-dependent Kinase inhibitor p21 as a major transcriptional target of p53, also serves as CDK inhibitor in G1 and S phase (Natsugoe et al., 1999). Among the target genes of the β-catenin/TCF complex, various cell growth related genes have been identified, among them Cyclin family is very important (Shtutman et al., 1999; Tetsu and McCormick, 1999) because the induction of Cyclin A/D1/E1 and B1 at the transcriptional level result in the progression of the G1/S phase and G2/M, respectively, as well as both increase the cell proliferation. Previous studies suggested that Cyclin D1 – CDK-4/6 and Cyclin A/E1 – CDK-2 complexes involve in hyper phosphorylation of pRb, which then activates E2F family induced cell proliferation (Deshpande et al., 2005). One recent study reported that polysaccharides from *Phellinus baumii* induced cell cycle arrest in various cancer lines in different phases, i.e., Hela and SGC-7901 in G0/G1 and RAW 264.7 in S phase (Liu M.M. et al., 2016). Our results showed that the significant change in the regulation of cell cycle related genes led to the polysaccharide induced cell cycle arrest Hela cell lines in respective phases. After analyzing our data, we hypothesized the proposed mechanism for the effects of prolific polysaccharide fraction (CASS) on cell cycle and apoptosis with a series of pathways involved in Hela cells (**Figure 5**).

Activation of the Caspase cascade (a family of cysteine proteases) plays a pivotal role in various apoptotic responses and lead to integral events in the apoptotic pathway. Caspase family consists of similar structures as well as possesses similar active sites containing cysteine residues which further selectively cut certain proteins and ultimately lead to cell death. The Caspase family members are mainly divided into two kinds, one kind for initiation and another for execution of apoptosis process. Moreover, PARP acts as an important indicator for Caspase 3 activation and also serves as vital marker for cell apoptosis (Lee et al., 2014). The mitochondria-mediated (intrinsic) apoptotic pathway is initiated by Caspase-9 followed by apoptosis execution by Caspase-3 which is activated by Caspase-9. Lee et al. (2014) reported that plant phytochemicals could decrease the expression level of anti-apoptotic proteins (Survivin, c-FLIP, Bcl-2) expression (Lee et al., 2014).

The Bcl-2 family of proteins plays critical roles in regulating cell death via apoptosis (Kodama et al., 2003). Bax/Bak pores on the outer mitochondrial membrane (Wang et al., 1997, 2001) take part in the release of Cytochrome C and other pro-apoptotic proteins into the cytosol, and further activate initiator Caspases-8 and -9, followed by the cleavage of Caspase-3, which ultimately triggers apoptosis (Lu et al., 2010).

Our findings have concluded that peony polysaccharides based on promotion of cell cycle arrest, apoptosis and inhibition of proliferation of different types of cancer cells *in-vitro* could be applied as potential anti-cancerous agent in various anti-cancerous treatments as key natural plant substances. Further, previous studies have also established a detailed relationship between different polysaccharides fractions in various cancer induced animal models. In brief, polysaccharides from *Lentinus edodes, Sargassum integerrimum*, Fucoidan and Lotus (*Nelumbo nucifera Gaertn.*) seeds showed anti-tumor activities under *in-vivo* conditions (Atashrazm et al., 2016; Liu G. et al., 2016; Zheng et al., 2016; Wang et al., 2017). One latest review updated the anti-cancerous potential of gynostemma polysaccharide in different normal and transgenic cancer mice models (Hsu et al., 2011; Lu et al., 2012; Liu et al., 2014; Tai et al., 2016). Thus, *in-vivo* studies using tested polysaccharide fractions at the next level would generate their comprehensive efficacy report for better prediction of their anti-cancerous potential.

## Conclusion

The focus of recent research has been shifted to pharmacologically active natural polysaccharides because of their importance in a wide range of oncology studies as well as their non-toxic characteristics. Our polysaccharide fractions previously reported to have remarkable physicochemical, rheological and antioxidant activities (Shi et al., 2016a,b). Further, to enhance their biological significance, the anti-cancerous effects of the four polysaccharide fractions (HBSS, CHSS, DASS, and CASS) obtained from peony seed dreg were studied. They consisted of different molecular weight, carbohydrate composition and their monosaccharide contents varied significantly which marked the effect of extraction methods on their chemical composition. Among the four fractions, CASS and DASS extracted by alkali method led to heterogenous monosaccharide composition which make this method an ideal for extracting various plant substances. Separation and purification of polysaccharides is very complicated, so it is quite difficult to obtain homogeneous active polysaccharide fractions. This is one of the main factors to impede polysaccharide research development. There are many methods and approaches for isolation, separation and purification of polysaccharides; researchers must carefully choose proper methods for separation and purification based on specific properties/characteristics of the polysaccharide to be researched CASS effectively inhibited the Hela cells followed by other cell lines as compared to 5-FU and led to cell cycle arrest in G0/G1 phase except MCF-7 cells and also the percentage of increased apoptotic cells differed in different treated cells. Excluding p53, CASS down regulated the Cyclin A/B1/D1/E1, CDK-1/2/4/6, and p15/16/21/27 in Hela cells. On the other hand, western blot analysis displayed remarkable change in expression of proteins (Cytochrome C, Bax, Bcl-2, *p*-Caspase-3, -8, -9, and PARP) were observed followed by Apaf-1 and Survivin in Hela cells. Taken together, our findings offer pre-clinical proof of anti-cancerous activities of peony polysaccharides based on promotion of cell cycle arrest, apoptosis and inhibition of proliferation of different types of cancer cells *in vitro*, and hence, CASS could be applied as potential anti-cancerous agent in chemotherapies in the near future and it can be further used in synergy with other polysaccharides under *in vivo* conditions by opting suitable animal models followed by clinical trials. Nevertheless, developing more efficient and economic ways for the preparation and modification of anti-cancerous polysaccharides and elucidating their structure-activity relationship remains significant challenge, and thus an active area of future research.

## Author Contributions

FZ was involved in the project design, carried out most of the experiments, and drafted the manuscript. J-JS participated in polysaccharide purification and molecular weight determination. KT cell culture analyzed the data and drafted the manuscript. FH and J-GZ participated in real time PCR and western blotting. Z-JW contributed substantially to the experimental design, manuscript preparation and submission. All authors read and approved the final manuscript.

## Conflict of Interest Statement

The authors declare that the research was conducted in the absence of any commercial or financial relationships that could be construed as a potential conflict of interest.

## Abbreviations

5-FU: Fluorouracil
CASS: Concentrated alkaline soluble solids
CCK-8: Cell Counting kit-8
CHSS: Chelating agent soluble solids
DASS: Dilute alkaline soluble solids
DMEM: Dulbecco’s Minimum Essential Medium
FBS: Fetal bovine serum
HBSS: Hot buffer soluble solids
LDH: Lactate dehydrogenase
McCoy’s 5A: McCoy’s 5A Modified Medium
PI: Propidium iodide
RPMI 1640: Roswell Park Memorial Institute Medium
UA: Uronic acid
