# Supplementary material for: Anti-Cancerous Potential of Polysaccharide Fractions Extracted from Peony Seed Dreg on Various Human Cancer Cell Lines Via Cell Cycle Arrest and Apoptosis
Source: Front Pharmacol. 2017 Mar 3;8:102. doi: 10.3389/fphar.2017.00102 (PMC5334287; doi:10.3389/fphar.2017.00102)

Anti-Cancerous Potential of Polysaccharide Fractions Extracted from Peony Seed Dreg on Various Human Cancer Cell Lines Via Cell Cycle Arrest and Apoptosis

Running Title: Anti-cancerous activities of peony polysaccharides

Fang Zhang[1], Jun-Jun Shi[1], Kiran Thakur[1], Fei Hu[1], Jian-Guo Zhang[1], Zhao-Jun Wei[1*]

[1] School of Food Science and Engineering, Hefei University of Technology, Hefei, People's Republic of China

* Correspondence:

Zhao-Jun Wei

zjwei@hfut.edu.cn

Supplementary Figure 1 Four types of peony seed dreg polysaccharides sequential extracted using hot buffer, chelating agent, dilute alkaline and concentrated alkaline.

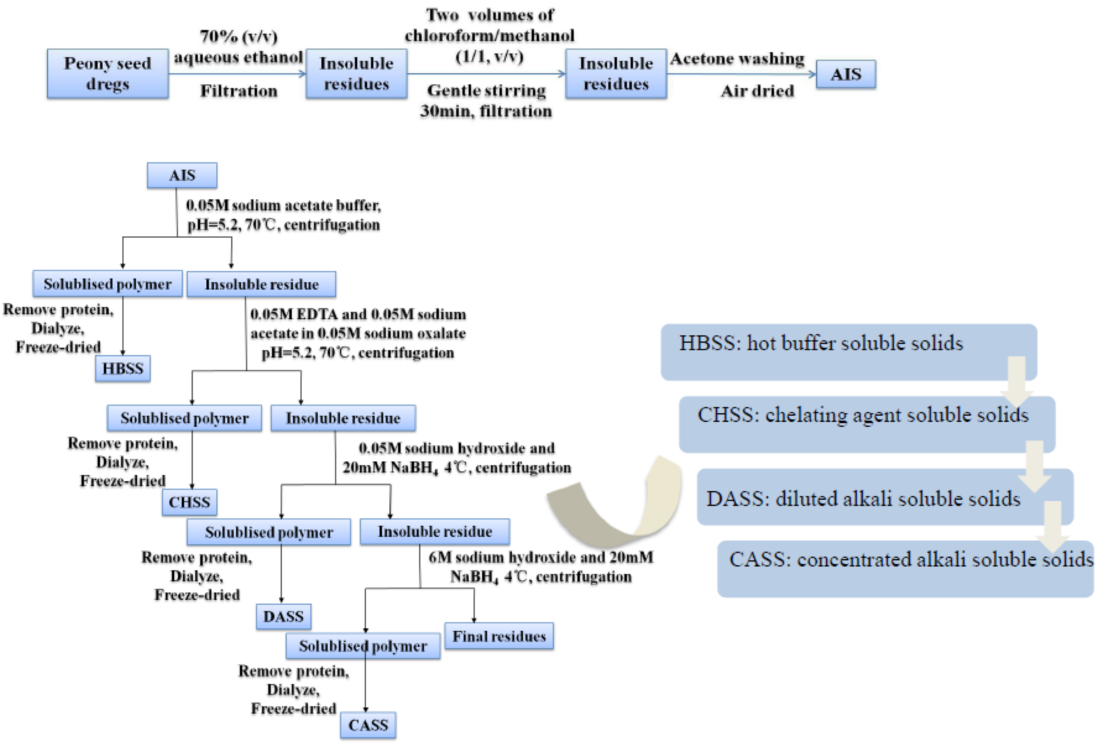

Supplementary Figure 2 Monosaccharide chromatograms of mixture standard and four peony seed dreg polysaccharides. Peak identity: 1. rhamnose; 2. arabinose; 3. xylose; 4. mannose; 5. glucose; 6. galactose.

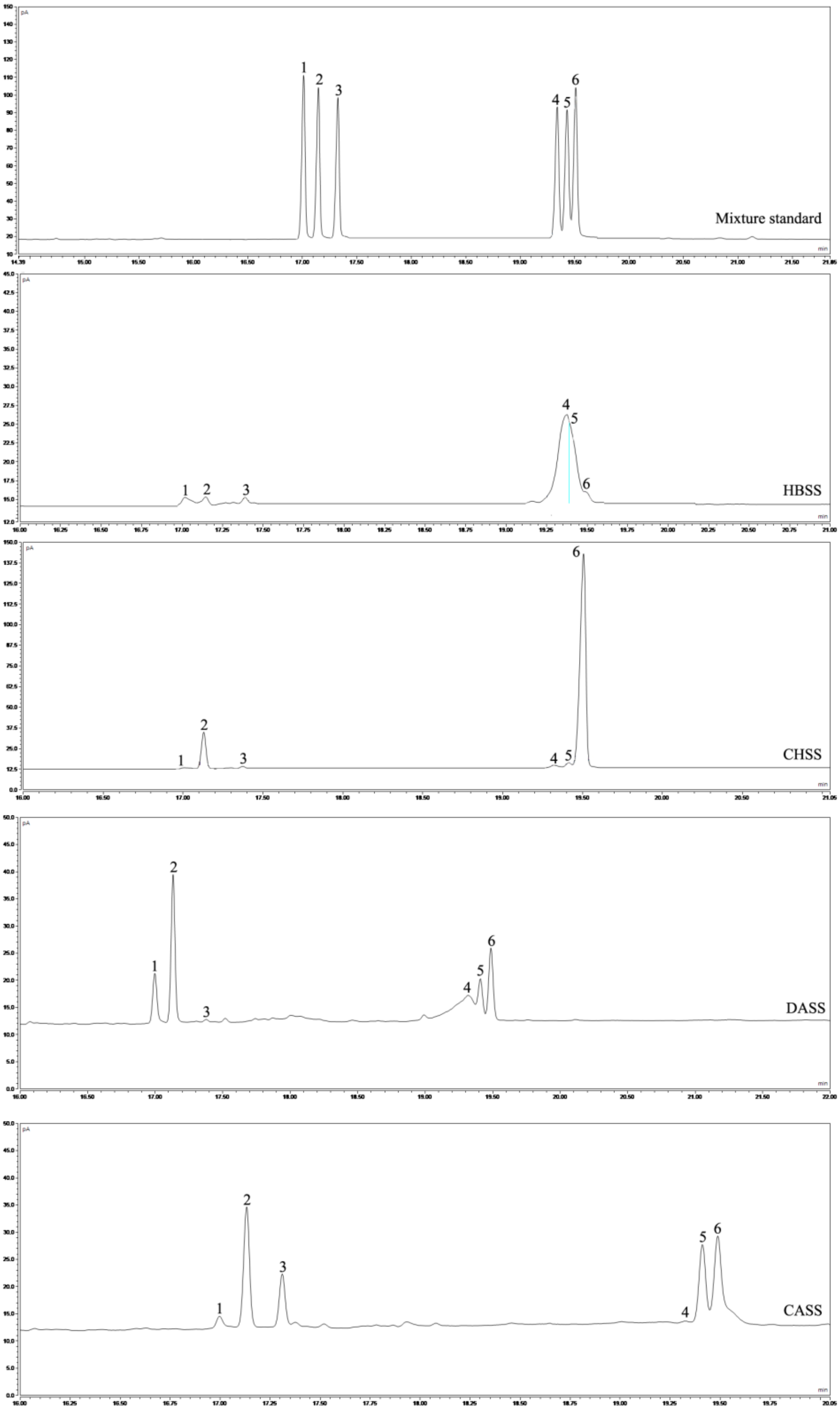

Supplement: Supplementary file 1 [file Image_1.PDF]
